# Distinct transcriptional modules in the peripheral blood mononuclear cells response to human respiratory syncytial virus or to human rhinovirus in hospitalized infants with bronchiolitis

**DOI:** 10.1371/journal.pone.0213501

**Published:** 2019-03-07

**Authors:** Sandra E. Vieira, Silvia Y. Bando, Milena de Paulis, Danielle B. L. Oliveira, Luciano M. Thomazelli, Edison L. Durigon, Marina B. Martinez, Carlos Alberto Moreira-Filho

**Affiliations:** 1 Department of Pediatrics, Faculdade de Medicina da Universidade de São Paulo, São Paulo, SP, Brazil; 2 Hospital Universitário da Universidade de São Paulo, São Paulo, SP, Brazil; 3 Department of Microbiology, Instituto de Ciências Biomédicas, Universidade de São Paulo, São Paulo, SP, Brazil; University of Georgia, UNITED STATES

## Abstract

Human respiratory syncytial virus (HRSV) is the main cause of bronchiolitis during the first year of life, when infections by other viruses, such as rhinovirus, also occur and are clinically indistinguishable from those caused by HRSV. In hospitalized infants with bronchiolitis, the analysis of gene expression profiles from peripheral blood mononuclear cells (PBMC) may be useful for the rapid identification of etiological factors, as well as for developing diagnostic tests, and elucidating pathogenic mechanisms triggered by different viral agents. In this study we conducted a comparative global gene expression analysis of PBMC obtained from two groups of infants with acute viral bronchiolitis who were infected by HRSV (HRSV group) or by HRV (HRV group). We employed a weighted gene co-expression network analysis (WGCNA) which allows the identification of transcriptional modules and their correlations with HRSV or HRV groups. This approach permitted the identification of distinct transcription modules for the HRSV and HRV groups. According to these data, the immune response to HRSV infection—comparatively to HRV infection—was more associated to the activation of the interferon gamma signaling pathways and less related to neutrophil activation mechanisms. Moreover, we also identified host-response molecular markers that could be used for etiopathogenic diagnosis. These results may contribute to the development of new tests for respiratory virus identification. The finding that distinct transcriptional profiles are associated to specific host responses to HRSV or to HRV may also contribute to the elucidation of the pathogenic mechanisms triggered by different respiratory viruses, paving the way for new therapeutic strategies.

## Introduction

Viral bronchiolitis is frequent and has an important impact on the children’s health care due to the high rates of hospitalization and mortality, especially of young infants [1, 2]. Human respiratory syncytial virus (HRSV) is the predominant etiological agent, but infections by other respiratory viruses, such as human rhinovirus (HRV), metapneumovirus, parainfluenza, influenza, adenovirus, and coronavirus, also occur. These infections with different respiratory virus present similar clinical characteristics, so etiological diagnosis can be carried out in clinical practice only by virus identification, either by molecular tests, immunofluorescence or culture methods [1–5]. Although the current guidelines do not indicate routine tests to identify the etiologic agent in infants with bronchiolitis, the etiological diagnosis may contribute to the prevention of nosocomial acquisition, since the transmission mechanisms diverge among respiratory viruses. Knowledge on molecular epidemiology also contributes to programming and organizing prophylactic strategies, such as the use of monoclonal antibodies to HRSV and influenza vaccination [3, 6]. Etiological diagnosis may also contribute for developing specific therapeutic approaches for each agent.

Clinical and epidemiological evidences indicated that pathogenic pathways are different in HRSV and HRV infections [7]. HRSV is the main agent of bronchiolitis, responsible for high rates of hospitalization and it is a major cause of mortality, especially in premature infants and those with risk factors. Despite this fact, the therapeutic approach consists mainly in supportive measures [3]. HRV is the most common agent of cold and triggering asthma attacks in atopic individuals, however around 35% of asymptomatic subjects have positive results for HRV tests [8]. Serious infections by both agents in early life are associated with recurrent wheezing in the following years, but this association is stronger with HRV. While HRSV infection leads to structural and functional changes in the airways, HRV infections do not cause as many changes and are more related to atopy and asthma [9]. Clinical studies suggest that the use of corticosteroids during the acute phase of infection with high levels of HRV may reduce the risk of recurrent wheezing in the subsequent year [10, 11].

Differential patient responses to respiratory viruses lead to different clinical outcomes and, interestingly, it has been found that infections with different respiratory viruses (HRSV, HRV, Influenza A), as well as with different genotypes of the same virus (HRSV), present distinctive PBMC transcriptome signatures [12] [13]. Furthermore, PBMC transcriptome profiles can be used to assess disease severity in infants with HRSV [7] and to predict individualized responses to HRV [14]. Thus, besides contributing to clarify the etiology, genomic methods can bring important information on the pathogenic role of the different respiratory virus as single agents, or in codetection, and in symptomatic and asymptomatic patients, which is especially important in HRV infections.

In this study, we conducted a comparative global gene expression analysis of PBMC obtained from patients with acute viral bronchiolitis infected by HRSV (HRSV group) or by HRV (HRV group). We employed a weighted gene co-expression network analysis (WGCNA) which allows the identification of transcriptional modules and their correlation with HRSV or HRV groups. This approach permitted the identification of distinct transcription modules for the HRSV and HRV groups. Moreover, differentially expressed genes in the PBMC expression profiles presented significant high fold-changes between HRSV and HRV groups and could be potential etiological markers.

## Materials and methods

### Ethics statement

This study was approved by the Research Ethics Committee of the Hospital Universitário da Universidade de São Paulo under number 1011/10. The infants’ legal guardians signed the Informed Consent Form after the presentation of the study by one of the authors and answered standardized questionnaires for the obtaining clinical, demographic and epidemiological data. All infants were submitted to molecular analysis of nasopharyngeal secretion for viral identification. Patients’ peripheral blood samples were collected for genomic analysis.

### Patient characteristics

A total of 12 out of 124 infants under 6 months of age—hospitalized between 2013 and 2015 at the Hospital Universitário da Universidade de São Paulo—were selected from a prospective cohort study on the etiology of acute viral bronchiolitis [15]. Diagnosis of bronchiolitis was defined as the first wheezing crisis, beginning no more than 3 days before hospital admission. In this study we included only infants infected with HRSV A ON1 or HRV as a single agent. Patients’ demographic and clinical data are listed in Table 1. The median age of the infants of the HRSV group and the HRV group were similar (41.5 days and 64 days, respectively; p = 0.8). There was a predominance of males in both groups (66.6% in the HRSV group and 83.3% in the HRV group). The mean length of hospital stay in the HRSV group was 6 days and in the HRV group was 2 days.

**Table 1:** Demographic and clinical data of infants with HRSV or HRV infection.

		days			Percentage			
Sample	Gender	Age	HS	WBC/mm3	LYMP	MNC	Neutrophils	Neutrophil/LYMP
RV46	Male	51	1	14000	81.0	7.0	12.0	0.15
RV66	Male	83	1	12210	57.0	10.0	33.0	0.58
RV81	Male	79	1	9830	67.8	9.1	23.1	0.34
RV109	Male	10	9	11010	43.0	11.0	46.0	1.07
RV116	Male	32	1	11120	67.3	14.6	18.1	0.27
RV117	Female	32	1	13090	68.3	13.3	18.4	0.27
**Median**		48	2*	11877*	64.1	10.8	25.1	0.45
RSV2	Female	31	4	11500	51.0	11.0	38.0	0.75
RSV6	Male	27	9	6080	66.0	12.0	22.0	0.33
RSV24	Male	88	5	6440	46.0	13.0	41.0	0.89
RSV32	Male	40	4	12000	84.0	6.0	10.0	0.12
RSV45	Male	130	6	8380	26.0	4.0	70.0	2.69
RSV48	Female	150	5	10400	53.0	8.0	39.0	0.74
**Median**		78	6*	9133*	54.3	9.0	36.7	0.92

HS–hospital stay; WBC—leukocytes; LYMP—lymphocytes; MNC—monocytes

*median significantly different between HRSV and HRV groups (t-test, p<0.05).

### Sample collection and total RNA extraction

Whole blood was collected in an EDTA-containing tube within a maximum of 24 hours after hospital admission. The peripheral blood mononuclear cells (PBMC) were immediately separated by gradient centrifugation using Ficoll-Paque Plus (GE Heathcare, cat. no. 17144002). After cell separation, the PBMC were collected, preserved in RNAlater (Qiagen, cat. no. 76106) and stored at -20°C until RNA extraction. PBMC were lysed with 300 μl of RLT buffer and the total RNA was extracted using RNeasy Mini Kit (Qiagen, cat. no. 74106). RNA purity analysis and quantification were performed using the NanoVue spectrophotometer (GE Heathcare Life Sciences, Marlborough, MA). RNA quality was assessed on the Agilent BioAnalyzer 2100 (Agilent, Santa Clara, CA). All samples presenting RIN ≥ 7.0 were stored at -80°C until used in hybridization experiments.

### Microarray hybridization and global gene expression

In order to determine gene expression profiles, 44 K DNA microarrays (Whole Human Genome Microarray Kit, Agilent Technologies, cat no. G4112F, Santa Clara, CA, USA) were used. The procedures for hybridization using the fluorescent dye Cy3 followed the manufacturer’s protocols (One-Color Microarray-Based Gene Expression Analysis—Quick Amp Labeling). The microarray images were captured by the reader Agilent Bundle, according to the parameters recommended for bio-arrays and extracted by Agilent Feature Extraction software version 9.5.3. Spots with two or more flags (low intensity, saturation, controls, etc.) were considered as NA, that is, without valid expression value. The R software version 2.11.1 and an in-house script were used for: i) excluding transcript spots presenting one or more NAs; iii) converting gene expression values to log base 2. Through this procedure the gene expression matrix with only expressed transcripts were obtained. Data normalization was performed using R software and the Lowess method [16]. TMEV software version 4.6.1 and t-test with p<0.05 and fold-change value ≥ 2.0 was used for obtaining the differentially expressed genes for the comparison (HRSV versus HRV). All microarray raw data have been deposited in Expression Omnibus (GEO) public database under accession number GSE124124.

### Weighted gene coexpression network analysis (WGCNA)

WGCNA is a method that identifies and characterizes gene modules whose members share strong coexpression. Networks were constructed using the WGCNA R software package [17]. Pearson’s correlation coefficient was used for obtaining gene coexpression similarity measures and for the subsequent construction of an adjacency matrix using soft power and topological overlap matrix (TOM). Soft-thresholding process transforms the correlation matrix to mimic the scale free topology. TOM is used to filter weak connections during network construction. Module identification is based on TOM and in average linkage hierarchical clustering. Finally, dynamic cut-tree algorithm was used for dendrogram’s branch selection. The module eigengene (ME) is defined as the first principal component of a given module, which can be considered a representative of the gene expression profiles in a module. Module Membership (MM), also known as eigengene-based connectivity (kME), is defined as the correlation of each gene expression profile with the module eigengene of a given module [17].

#### Module-HRSV or HRV association

Firstly, we obtained the gene significance (GS), which is a value of the correlation between the trait (here is represented by HRSV or HRV groups) and the gene expression values. The mean GS for a particular module is considered as a measure of module significance (MS). The GS values were obtained using Pearson’s correlation and to assign a *p*-value to the module significance, we used Student’s t test. The modules, which presented high positive correlation value with HRSV or HRV groups (r ≥ 0.6 and p < 0.05) were selected for biological functional analysis.

#### Intramodular analysis for hub selection

The MM and GS values were used for gene categorization. Genes presenting high GS and MM were considered as hubs in the module and significantly associated with HRSV or HRV groups. We plotted all gene values in a MM (x-axis) vs GS graphic (y-axis).

### Functional enrichment analysis

KEGG pathway enrichment was performed using Enrichr online web-based tool [18] to analyze the network modules that are associated with the HRSV or HRV groups (p < 0.05). The biological function analysis for the selected genes was done using the Gene Ontology database.

## Results

Transcriptional analysis revealed that 283 out of 6,615 GO annotated genes were differentially expressed (DE, fold-change ≥ 2.0) when compared between the HRSV and HRV groups. In the HRSV group most of the DE genes (204 genes) were hyper-expressed and only 79 genes were hypo-expressed when compared with the HRV group. The gene *CCDC177* (*C14orf162*) presented the highest fold-change value (16.3). Moreover, 22 genes were differentially expressed and presented high fold-change values between 16.3 and 4.0 (S1 Fig). Table 2 lists these high fold-change DE genes, where 21 genes were hyper-expressed and one was hypo-expressed in HRSV group.

**Fig 1:**
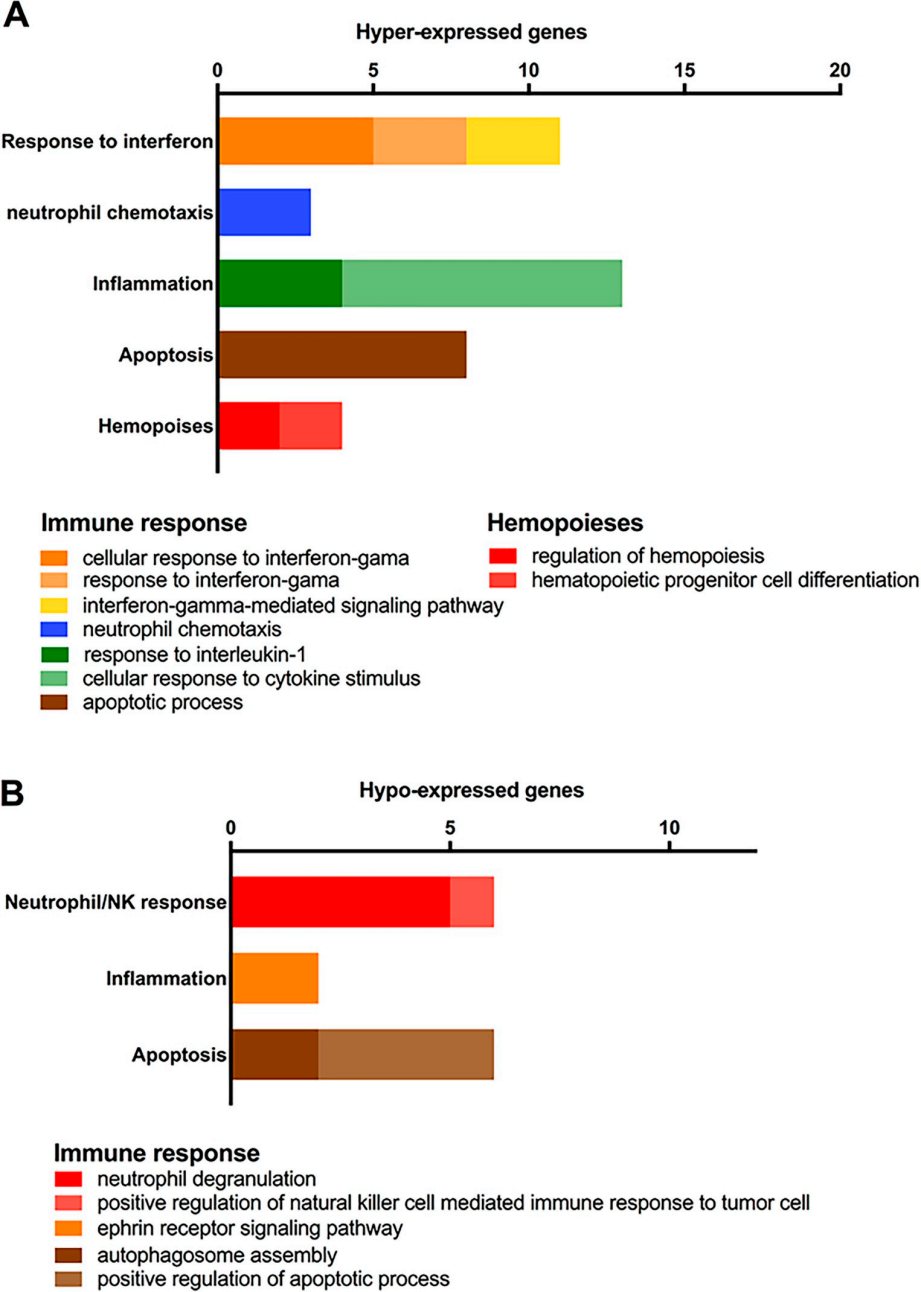
Enrichment analysis for DE genes. Gene categorization and distribution according to the terms listed for Biological Process in the Gene Ontology database (p<0.05). Hyper- (**A**) and hypo-expressed (**B**) genes in the HRSV group.

**Table 2:** Differentially expressed genes with high fold-change (>4.0) for HRSV group in comparison with HRV group.

Gene	Gene expression average		FC*	Adj *p*	Functional description
	HRSV group	HRV group	HRSV/HRV		
*C14orf162*	2602.4	160.0	16.3	0.02	aliase *CCDC177*; coiled-coil domain containing 177
*ZDHHC20*	556.4	66.1	8.4	0.00	palmitoyltransferase activity
*FAM118A*	6773.3	999.9	6.8	0.00	protein binding
*TTC28*	2678.2	401.9	6.7	0.00	cell division
*ZNF2*^a^	6052.0	910.6	6.6	0.00	regulation of transcription, DNA-templated
*ZSCAN2*	3203.1	482.2	6.6	0.00	regulation of transcription, DNA-templated
*POLR2J2*	3675.9	587.7	6.3	0.00	transcription, DNA-templated
*TBXA2R*	4479.4	759.7	5.9	0.00	G-protein coupled receptor signaling pathway; inflammatory response
*KIAA1875*	13424.5	2329.1	5.8	0.00	aliase WDR97; WD repeat domain 97; function unkhown
*COX6B2*	22173.3	4028.8	5.5	0.00	protein binding; mitochondrial crista
*LOC284454*	2120.3	412.8	5.1	0.01	ncRNA
*SOX13*	509.6	102.7	5.0	0.01	DNA-binding transcription factor activity
*LAG3*	408.5	82.7	4.9	0.00	MHC class II protein binding; positive regulation of natural killer cell mediated cytotoxicity
*HYAL4*	7786.2	1615.4	4.8	0.00	glycosaminoglycan catabolic process
*ZNF713*	9430.2	1961.7	4.8	0.01	regulation of transcription, DNA-templated
*EVX1*^b^	2108.4	445.8	4.7	0.00	regulation of transcription, DNA-templated
*SERPING1*	148.5	32.4	4.6	0.04	complement activation, classical pathway
*WDR90*	1029.3	244.6	4.2	0.00	WD repeat domain 90; protein binding
*LGALS3BP*^b^	1167.2	286.0	4.1	0.00	cellular defense response; receptor-mediated endocytosis
*MOCS3*	4127.3	1026.0	4.0	0.00	Mo-molybdopterin cofactor biosynthetic process
*AMN*	966.0	242.7	4.0	0.00	receptor-mediated endocytosis
*SIRPB1*	109.8	607.9	-5.5	0.02	neutrophil degranulation

^a^gene also is HGS-hub of the HRV group

^b^genes also are HGS-hub of the HRSV group

*FC, fold-change was calculated by the ratio of gene expression median in HRSV/HRV groups. The comparison was done using t-test with adjusted Bonferroni correction (p<0.05 was considered significant).

The enrichment analysis of the DE genes, based on Biological Process (BP) terms of Gene Ontology database, showed that 99 DE genes (60 hyper-expressed and 39 hypo-expressed genes in the HRSV group in comparison with the HRV group) are significantly over-represented in BP terms. Twenty-one hyper-expressed genes are involved in immune response processes, such as inflammatory response, apoptosis, response to interferon-γ, and neutrophil chemotaxis. Thirteen hypo-expressed genes are also related to immune response, involving neutrophil degranulation, inflammatory response, apoptosis and phagocytosis (Fig 1; S1 and S2 Tables).

### WGCNA

**Fig 2:**
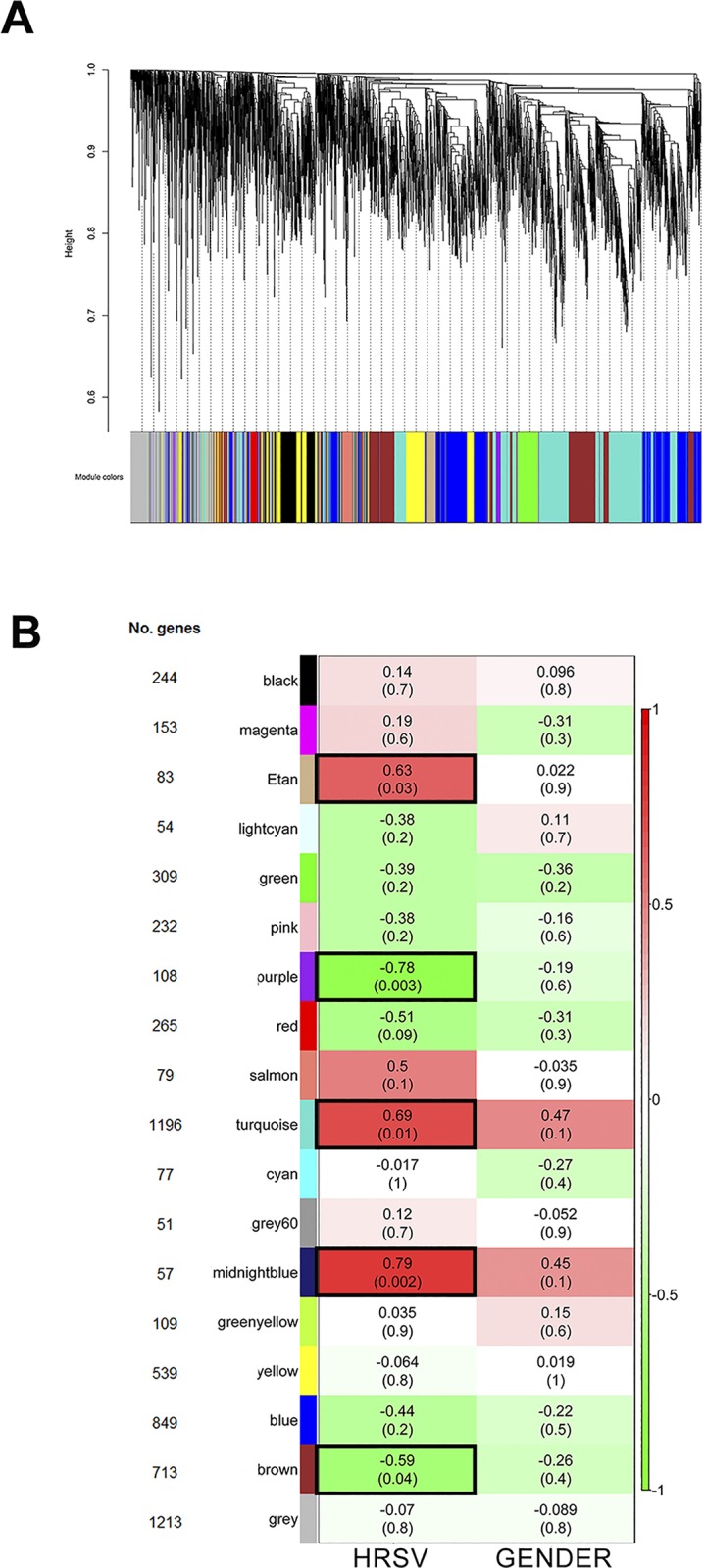
WGCNA dendrogram and modules correlation with HRSV group and gender. **A**- Hierarchical clustering dendrogram and module identification, indicated by different colors; **B**- Heatmap of the relationship between modules (MEs) and traits (infection and gender). Only the HRSV group is shown. Numbers inside each colored box are the correlation coefficients between the ME and the specific trait, with p-value between brackets. The same values are true for the HRV group, but with an opposite correlation coefficient signal. The more intense the color of the box, the more negatively (green) or positively (red) correlated is the module with the trait. Five modules presented significant association (p<0.05) with the HRSV group and these modules are indicated by black borders.

The coexpression network was constructed by Pearson’s correlation between all genes and considering a soft-thresholding power β of 22, resulting in a scale-free network. Following dynamic tree cut, the hierarchical clustering dendrogram identified 17 distinct gene modules (Fig 2). Modules are defined as branches of the network dendrogram and identified by different color. Modules size ranged from 51 (grey60 module) to 1,196 (turquoise module) genes. Genes not classified in any correlated module were grouped in a grey module.

After the modules were generated, HRSV and HRV groups were then correlated with the modules. A total of five modules were significantly (p<0.05) associated with HRSV. Three of those five modules were positively associated with HRSV group: midnight blue (MS = 0.79, *p* = 0.002), turquoise (MS = 0.69, *p* = 0.01) and tan (MS = 0.63, *p* = 0.03) modules. Others two modules were positively associated with HRV group: purple (MS = 0.78, *p* = 0.003) and brown (MS = 0.59, *p* = 0.04) modules. None module was associated with gender (Fig 2B).

**Fig 3:**
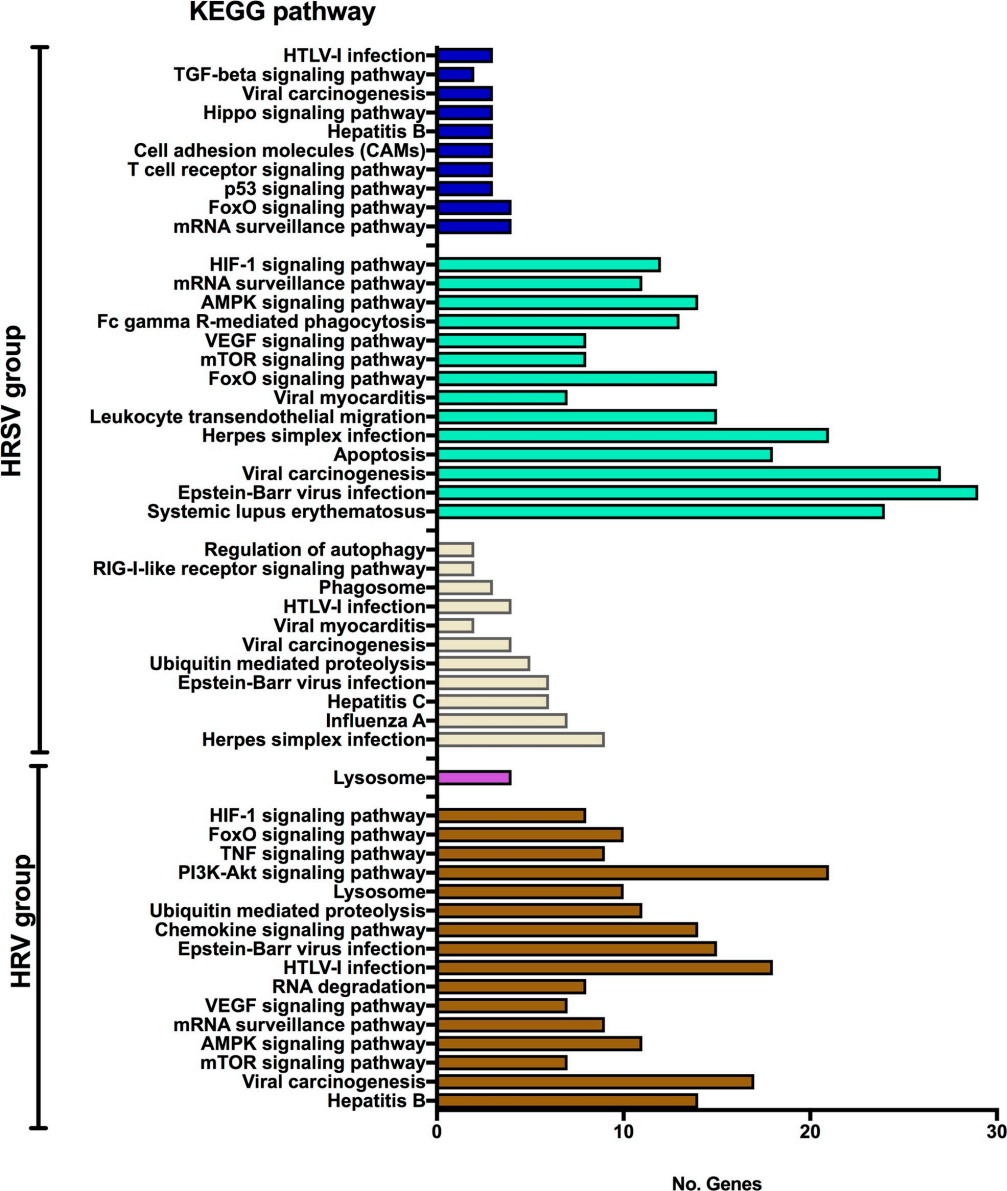
KEGG enrichment analysis of significantly associated modules with HRSV or HRV groups. Midnight blue (MS = 0.79, *p* = 0.002), turquoise (MS = 0.69, *p* = 0.01) and tan (MS = 0.63, *p* = 0.03) are modules positively associated with HRSV. Purple (MS = 0.78, *p* = 0.003) and brown (MS = 0.59, *p* = 0.04) are modules positively associated with HRV. The bar colors correspond to each module.

The functional profiles of these five gene modules—based on enrichment analysis using KEGG pathways database—showed that the midnight blue and tan modules (positively associated to HRSV) contain a high proportion of genes (about 60%) involved in cell signaling and in immune response to viral infection. Conversely, the turquoise (positively associated to HRSV), brown, and purple modules (positively associated to HRV) have relatively few genes (26% in brown, 19% in turquoise, and 3.7% in purple) involved in cell/ immune response to viruses (Fig 3). A complete list of KEGG pathways found for these modules are listed in S3 Table.

**Fig 4:**
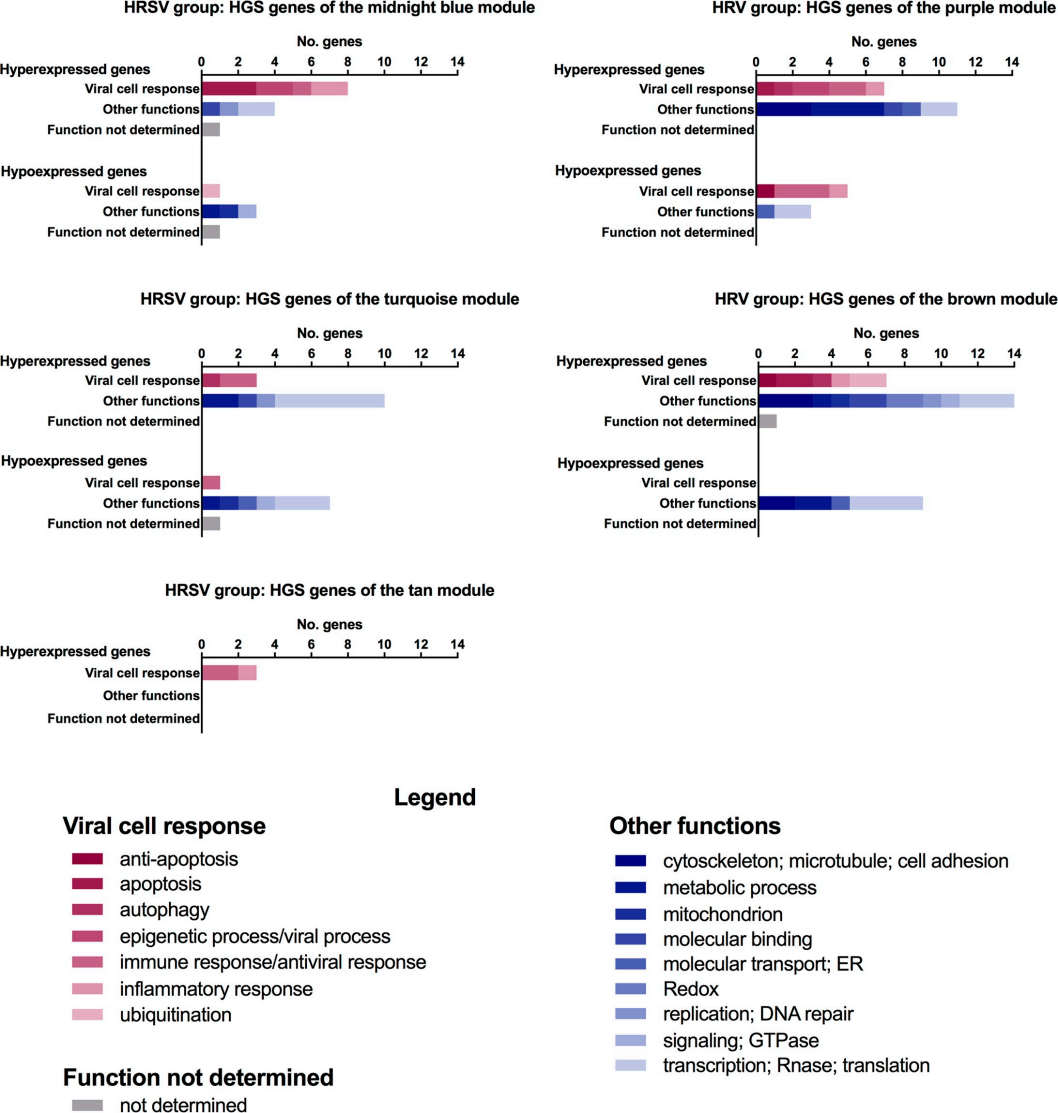
Functional profile of the HGS-hub genes. Biological function categorization and distribution of HGS-hubs in the five modules positively associated with HRSV or HRV groups.

Then, we categorized the genes of the five modules positively associated with HRSV (midnight blue, turquoise, and tan) or with HRV (brown and purple) considering module membership (MM) and gene significant (GS) values for HRSV or HRV groups (S2 Fig). We identified a total of 42 and 57 genes with highest GS and MM values (GS and MM values ≥ 0.75; p < 0.01 was considered significant; S4 and S5 Tables) for HRSV and for HRV groups, respectively. These genes were named here as HGS-hubs. The biological function of the HGS-hubs in HRSV and HRV groups is listed in Tables 3 and 4, respectively. Functional and gene expression profiles of the HGS-hubs and their distribution in the modules of HRSV and HRV groups are depicted in Fig 4.

**Table 3:** Functional description of the HGS-hub genes in modules highly and positively associated with HRSV group.

ME	Gene	FC^a^	GO terms or PubMed	Biological function
Midnight blue	*CCNA2*	2.62	positive regulation of transcription, DNA-templated; viral process	transcription/viral process
	*MED6*	1.19	regulation of transcription by RNA polymerase II	transcription
	*PPP2R1B*	1.36	positive regulation of extrinsic apoptotic signaling pathway in absence of ligand	apoptosis
	*PGRMC2*	1.39	protein binding	protein binding
	*CD28*	1.51	positive regulation of inflammatory response to antigenic stimulus	inflammatory response
	*TPX2*	2.20	apoptotic process	apoptosis
	*HIST1H1B*	2.03	histone deacetylase binding	transcription
	*FEN1*	1.67	double-strand break repair	replication
	*CCNB1*	1.81	histone H3-S10 phosphorylation involved in chromosome condensation	epigenetic process
	*PDCD1*	1.70	positive regulation of T cell apoptotic process	apoptosis
	*KIF23*	1.90	antigen processing and presentation of exogenous peptide antigen via MHC class II	immune response
	*CXCR6*	2.42	chemokine-mediated signaling pathway; inflammatory response	inflammatory response
	*NPEPL1*	0.74	aminopeptidase activity	metabolic process
	*SLC25A16*	0.57	mitochondrial transport	mitochondrion
	*LOC388242*	0.43	pseudogene	ND
	*TSPAN32*	0.60	cell surface receptor signaling pathway	signaling
	*ANKRD13D*	0.73	late endosome [19]	ubiquitination
Turquoise	*PFDN1*	1.17	regulation of transcription, DNA-templated	transcription
	*CSNK2B*	1.29	macroautophagy; neutrophil degranulation	autophagic process
	*DCST2*	3.89	codifies a dendritic cell-specific transmembrane protein	immune response
	*LGALS3BP*	4.23	cell adhesion; cellular defense response; receptor-mediated endocytosis	immune response/antiviral response
	*FAM48A*	1.82	alias *SUPT20H*; regulation of transcription by RNA polymerase II	transcription
	*YWHAQ*	1.49	negative regulation of transcription, DNA-templated	transcription
	*HIST1H2AM*	1.63	chromatin organization	transcription
	*B4GALT2*	2.18	galactosyltransferase activity	metabolic process
	*TMEM160*	1.57	integral component of membrane	protein binding
	*HIST2H2AC*	1.83	chromatin organization	transcription
	*RNASEH2A*	1.58	DNA replication	replication
	*EVX1*	4.89	positive regulation of transcription from RNA polymerase II promoter	transcription
	*PFKP*	1.29	glucose catabolic process	metabolic process
	*CCDC84*	0.70	coiled-coil domain protein	ND
	*EFNB1*	0.43	ephrin receptor signaling pathway; T cell costimulation	immune response
	*BTAF1*	0.62	negative regulation of transcription, DNA-templated	transcription
	*TRIT1*	0.62	mitochondrial tRNA modification	mitochondion
	*DENND4B*	0.63	regulation of Rab protein signal transduction	signaling
	*ZMIZ2*	0.59	positive regulation of transcription by RNA polymerase II	transcription
	*RNASEH2C*	0.65	RNA catabolic process	Rnase
	*C19orf6*	0.54	aliase *TMEM259*; codifies an aspecific BCL2 ARE-binding protein 1	ER
	*GUSB*	0.69	carbohydrate metabolic process	metabolic process
Tan	*TRAFD1*	1.60	response to cytokine	inflammatory response
	*APOL1*	2.09	cytolysis; killing of cells of other organisms	immune response/antiviral response
	*IDO1*	3.81	NOT regulation of activated T cell proliferation	immune response

^a^FC, HRSV/HRV fold-change >1.0 –hyper-expressed genes or < 1.0—hypo-expressed genes

ME–module eigengene.

**Table 4:** Functional description of the HGS-hub genes in modules highly and positively associated with HRV group.

ME	Gene	FC^a^	GO terms or PubMed	Biological function
Purple	*STK11IP*	1.54	neutrophil degranulation	immune response
	*PPP1R16A*	1.54	regulation of phosphoprotein phosphatase activity	metabolic process
	*FRY*	1.99	microtubule organizing center	microtubule
	*TPPP3*	2.80	microtubule bundle formation	microtubule
	*EFEMP2*	2.09	calcium ion binding; extracellular matrix organization	matrix organization
	*KCNQ1*	1.77	calmodulin binding	ion transport
	*RASSF2*	1.36	negative regulation of NIK/NF-kappaB signaling; positive regulation of apoptotic process	apoptosis
	*ZNF562*	1.30	regulation of transcription, DNA-templated	transcription
	*CHP*	1.48	negative regulation of NF-kappaB transcription factor activity	immune response
	*MSRA*	1.46	response to oxidative stress	metabolic process
	*RCOR1*	1.76	histone H4 deacetylation; viral process	transcription/viral process
	*UHRF1BP1*	1.56	histone deacetylase binding	epigenetic process
	*WDFY2*	1.67	positive regulation of protein phosphorylation	ion binding
	*H3F3A*	1.28	histone binding	transcription
	*CCDC88A*	1.83	TOR signaling	autophagic process
	*GBE1*	1.70	glucose metabolic process	metabolic process
	*CBL*	2.12	ephrin receptor binding	inflammatory response
	*SLC16A5*	1.54	monocarboxylic acid transport	metabolic process
	*TNFRSF4*	0.57	positive regulation of T cell cytokine production	inflammatory response
	*SLC38A5*	0.48	amino acid transport	protein transport
	*IGSF8*	0.70	protein binding; cell motility; immunoglobulin protein superfamily; cell migration and viral infection	immune response/antiviral response
	*FTSJ3*	0.79	rRNA methyltransferase activity	transcription
	*HAX1*	0.81	interleukin-1 binding; negative regulation of apoptotic process	anti-apoptosis
	*CCDC101*	0.70	histone H3 acetylation; SAGA complex; SAGA complex mediates the transcriptional up-regulation of antiviral RNA silencing [20]	antiviral response
	*PSMC4*	0.85	antigen processing and presentation of peptide antigen via MHC class I; apoptosis	immune response
	*MRPL49*	0.73	mitochondrial translation	translation
Brown	*ZDHHC8*	1.66	regulation of mitochondrion degradation	mitochondrion
	*PTAR1*	1.59	protein prenylation	metabolic process
	*FAM122B*	1.50	ND	ND
	*VHL*	1.51	negative regulation of apoptotic process; ubiquitin protein ligase activity	anti-apoptosis
	*TSC1*	1.85	GTPase regulator activity; cell-matrix adhesion	cell adhesion
	*IFT172*	1.54	Notch signaling pathway	inflammatory response
	*PACS2*	2.74	autophagosome assembly; apoptotic process	autophagic process
	*SLTM*	1.71	apoptotic process	apoptosis
	*ASCC1*	1.31	DNA repair	DNA repair
	*SLC12A6*	1.73	protein kinase binding	protein binding
	*HIPK1*	1.74	extrinsic apoptotic signaling pathway	apoptosis
	*TMEM107*	1.46	cilium assembly	cytoskeleton/cilium
	*REEP5*	1.35	protein binding	protein binding
	*TBC1D9*	1.84	GTPase activator activity	GTPase
	*CROCC*	1.39	actin cytoskeleton	cytoskeleton/cilium
	*MLL*	1.44	chromatin modifying enzymes	transcription
	*CBR4*	1.74	oxidation-reduction process	Redox
	*TMEM129*	1.70	ubiquitin protein ligase activity	Ubiquitin
	*POP1*	1.34	tRNA processing	translation
	*SNAPC4*	1.46	chromatin binding	transcription
	*ZADH2*	1.58	oxidation-reduction process	Redox
	*FBXO41*	1.62	ubiquitin-protein transferase activity	Ubiquitin
	*CTNNB1*	0.83	transcription elongation from RNA polymerase II promoter	transcription
	*DYNLRB1*	0.68	SMAD binding; cell-matrix adhesion	cell adhesion
	*ZDHHC24*	0.77	microtubule motor activity	microtubule
	*GTF2B*	0.72	regulation of transcription, DNA-templated	transcription
	*ZFR*	0.80	pyruvate metabolic process	metabolic process
	*ZNF2*	0.15	protein transmembrane transport	protein transport
	*TIMM17B*	0.75	protein-cysteine S-palmitoyltransferase activity	metabolic process
	*ADRM1*	0.76	poly(A) RNA binding	translation
	*PDHX*	0.71	transcription, DNA-templated	transcription

^a^FC, HRV/HRSV fold-change >1.0 –hyper-expressed genes or < 1.0—hypo-expressed genes

ME–module eigengene.

In the HRSV group, 16 out of 46 HGS-hubs were related to immune and inflammatory responses, phagocytosis and apoptosis (Table 3). Moreover, the majority of these HGS-hubs is hyper-expressed in the HRSV group. It’s interesting to note that 11 out of 28 HGS-hubs had fold-change values > 2.0. Seven of these genes are involved in immune responses: six are hyper-expressed (*TPX2*, *CXCR6*, *DCST2*, *LGALS3BP*, *APOL1*, and *IDO1*); and one (*EFNB1*) is hypo-expressed.

In the HRV group, comparatively to the HRSV group, just a small number (19 out of 71 genes) of HGS-hubs are involved in cell response to virus and majority of them is hyper-expressed in the HRV group (Table 4). Three hyper-expressed HGS-hubs are involved in immune response signaling pathways, such as negative regulation of NF-KappaB kinase signaling pathway (*RASSF2* and *CHIP*) and TOR signaling pathway (*CCDC88A*). Only two HGS-hubs, *CBL* and *PACS2*, had fold-change values > 2.0 and are involved in inflammatory response and autophagic process, respectively.

## Discussion

Bronchiolitis is frequently caused by HRSV but HRV is also an important etiological agent. Infections by these two viruses present similar clinical features, thus rendering etiological diagnosis difficult. Moreover, no vaccines or effective/specific therapies are available. The identification of the etiology in infant bronchiolitis by obtaining peripheral blood samples and performing molecular marker analyses would be of great utility in clinical practice. These methods are non-invasive and reflect quite well the host's immunological response [21], thus allowing a better understanding of the molecular mechanisms involved in the pathogenesis of HRSV infection.

Previous epidemiological and genotypical studies have indicated that the diseases caused by HRSV and HRV display some differences [4]. Infants, especially under 6 months of age, are more susceptible to hospitalization and death in HRSV infections [1, 2]. Rodriguez-Fernandez et al [13] showed that infants with bronchiolitis presented different transcriptome profiles for distinct HRSV genotypes. For instance, the GA5 genotype was associated with diminished interferon activation and increased expression of genes involved in neutrophil activation, comparatively to other HRSV genotypes. Considering these facts, we selected infants under 6 months of age, who had a first episode of wheezing, and only infants with single infection with HRSV A ON1 genotype.

In this study, we found two distinct PBMC transcriptomic profiles and the differentially expressed genes subsets are related to different innate immune response of the patients with HRSV or HRV bronchiolitis. Mejias et al. [7] also showed a specific gene expression profile in infants infected with HRSV, characterized by overexpression of innate immunity and suppression of adaptive immunity, and associated with neutrophil response, inflammation, IFN activation and suppression of T and B cell genes. Here is important to recall that HRSV infects PBMCs during the immune response to viral challenge, when these cells are recruited to the respiratory trait, and differential immune response to HRSV is determined early after exposure [22].

Additionally, the leukogram profile (Table 1) showed significantly (Mann-Whitney test, p = 0.03) more leukocytes in infants with HRV infection than in infants with HRSV infection. Choi et al [23] also found leukocytosis in infants with HRV infection compared with other respiratory viruses (HRSV, influenza virus, parainfluenza virus, human coronavirus, human bocavirus, human metapneumovirus). However, all infants studied here had neutropenia (neutrophil/lymphocyte ratio ≤ 1.0) and no significant difference between HRSV and HRV groups was found. It’s interesting to mention that Choi et al [23] found a different neutrophil count, which neutrophil/lymphocyte ratio was over than 3.0 for HRV and less than 1.0 for HRSV infection. This neutropenia observed in both group of infants in our study probably was caused by neutrophil migration to lung as previously observed by Everard et al [24] analyzing bronchial secretion of children with HRSV infection.

It is well established that systemic neutrophil response correlates with increased disease severity and it is mediated by the neutrophil chemoattractant. In the fatal cases of HRSV inflammatory neutrophils were found widespread in lung tissue [25]. Moreover, Choi et al [23] showed that HRV is associated with neutrophil activation and inflammatory response while HRSV is associated with lymphocyte-mediated inflammation. In this study, the PBMC transcriptomic analysis revealed different expression profiles between HRSV and HRV infection. Five hypo-expressed genes in HRSV group—*SYNGR1*, *AGL*, *LAMP2*, *ORM2*, and *SIRPB1* –are involved in neutrophil activation processes, while three genes expressing neutrophil chemoattractants—*CCL15*, *CCL24*, and *LGALS3*—were hyper-expressed in HRSV infection (S1 and S2 Tables). Our results indicated that the host response to HRSV, compared with HRV, are related to diminished neutrophil activation and increase neutrophil chemotaxis. This response could well be associated to disease severity and, consequently, to longer periods of hospitalization (Table 1).

Another subset of hyper-expressed genes—*OAS2*, *PTAFR*, *PML*, *MEFV*, *CCL15*, and *CCL24*—in HRSV group is related to interferon-gamma pathway (S1 Table). Interferon induces antiviral effectors encoding genes and exerts a protective effect [25, 26]. Moreover, the balance between pro- and anti-inflammatory T-cell cytokine responses may determine the clinical outcome of HRSV infection. High levels of IFN-gamma might reduce viral replication but increase immunopathology. However, decreased IFN-gamma response has been reported to be associated with severe HRSV infection [27]. The treatment with systemic steroids is controversial regarding the efficacy of systemic steroid use in severe bronchiolitis. Pinto et al [28] showed that infants with severe illness had higher plasma cortisol levels than infants with mild disease, and a decreased IFN-gamma production by PBMCs in severely affected infants. Nevertheless, these two molecular mechanisms—neutrophil chemotaxis and IFN-gamma/cortisol–seem to be important therapeutic targets for modulating HRSV-derived immunopathologies.

The gene coexpression network analysis revealed distinct modules (tan, midnight-blue, turquoise, purple and brown) containing genes involved in immune response, activation of interferon pathway and apoptosis. Module analysis identified HGS-hubs associated with HRSV and HRV groups. In network, hubs are important to maintain the structure of the network or modules [29–31]. Additionally, high gene significant (HGS) value implies that the gene is significantly associated to phenotypic/genotypic features of the group [17], i.e., HRSV or HRV infections and the gene is differentially expressed between groups. Therefore, these HGS-hubs might be used to differentiate these two etiologies.

Several HGS-hubs in both groups (14 out of 42 in HRSV group and 14 out of 57 in HRV group, Tables 3 and 4) are involved in immune response but the molecular mechanism differs between HRSV and HRV groups: the HGS-hubs involved in inflammatory response, apoptosis, and autophagy are not the same in each group. It’s is interesting to mention that nine HGS-hubs had fold-change > 2.0 and are involved in immune response, inflammatory, and apoptosis. Moreover, eight of these genes might be potential therapeutic/vaccine targets and/or molecular markers, as commented below.

Six out of eight HGS-hubs were previously described as potential therapeutic targets: i) *LGALS3BP* encodes for galectin 3 binding protein, an antiviral protein that interferes with human immunodeficiency virus type 1 replication diminishing the virions infectivity [32]; ii) *EFNB1* codifies an ephrin-B1; members of Efns family are expressed in thymocytes and T cells and they are capable to modulate T cell responses and survival contributing to the integrity of an immune response [33]; iii) *APOL1*, codifies for an apolipoprotein 1, which is a mediator protein acting on IFN-activated genes [34, 35] and the proteins of the apolipoprotein family, such as APOL6, has an HRSV antiviral activity related to apoptosis [36]; iv) *IDO1* encoded protein was described to control viral infection by modulating specific metabolic events [37]. IDO1 was hyper-expressed after IFN-mediated induction in response to Influenza A viruses and this enzyme is involved in cytokine overproduction and T cell suppression [38]; v) *CBL*, alias *C-CBL*, encodes for RING finger E3 ubiquitin ligase and it is involved in antiviral process by activation IFN-I pathway [39]; and vi) *PACS2*, that codifies for a sorting protein PACS-2, interacts with a protein Nef of the human immunodeficiency virus type 1 [40]. This interaction mediates the MHC-I down-regulation and consequently the virus escape from host immunity [40].

Another strategy to combat HRSV could be a vaccine therapy. We identified one HGS-hub that might be a potent vaccine target: *CXCR6*, that codifies for a chemokine expressed by natural killer T (NKT) cells and it’s essential for NKT cells trafficking [41]. It was described that the activation of NKT cells by intranasal coadministration of α-galactosylceramide, in a nasal vaccine against influenza, can potently enhance protective immune responses through increasing NKT cell population in nasal mucosa [41].

Finally, two HGS-hubs could be potential molecular markers for HRSV infection. *LGALS3BP*, which is also a potential therapeutic target [32], and the other is *DCST2* that codifies for a dendritic cell-specific transmembrane protein and its intracellular localization is due in response to toll-like receptor ligation [42]. These genes presented high fold-changes and were hyper-expressed in PBMC from infants infected with HRSV. Additionally, 22 DE genes had higher fold-changes (≥ 4.0) and six of them (one is the HGS-hub *LGALS3BP*) are involved in immune response, and other two genes related to transcriptional regulation are HGS-hubs (Table 2). Finally, the gene *CCDC177* codifying a coiled-coil domain protein presented the highest fold-change 16.3.

## Conclusions

The PBMC transcriptional profiles of hospitalized infants with HRSV or HRV bronchiolitis are different and probably correlated with distinctive etiopathogenic mechanisms. Additionally, the infants with HRSV had leukopenia and they usually demand more hospitalization days. The immune response to HRSV infection, comparatively to the response to HRV infection, appears to be more associated to the activation of the IFNγ signaling pathways and less related to neutrophil activation. Moreover, we also identified potential host-response molecular markers that could be used for HRSV or HRV etiopathogenic diagnosis. These results may contribute to the development of future tests for respiratory virus identification. Additionally, a better understanding of PBMC specific host responses to HRSV or HRV—here disclosed by different gene expression profiles—may serve to elucidate the pathogenic mechanisms triggered by different viral agents and, therefore, contribute to the development of new therapeutic approaches.

## Supporting information

S1 Fig Fold-change value distribution for 283 differentially expressed (DE) genes (HRSV x HRV). Twenty-two genes were identified as candidate gene markers. The red dot line indicates the cut off (fold-change 4.0) adopted here to consider highly differentially expressed genes.
(TIF)

S2 Fig HGS-hub categorization for HRSV and HRV groups. Scatterplots between MM (x-axis) and GS (y-axis) of the genes in the i) midnight blue, turquoise, and tan modules of the HRSV group; ii) brown and purple modules of the HRV group. The red or blue dot lines indicate, respectively, the cut off of GS or MM values significantly for HRSV or HRV groups (p < 0.01).
(TIF)

S1 Table Enrichment analysis for hyper-expressed genes in HRSV group. (XLSX)

S2 Table Enrichment analysis for hypo-expressed genes in HRSV group. (XLSX)

S3 Table KEGG pathway enrichment analysis of the network modules associated with the HRSV or HRV groups (p<0.05 was considered significant). (XLSX)

S4 Table HGS-hubs in modules highly and positively associated with HRSV group. (XLSX)

S5 Table HGS-hubs in modules highly and positively associated with HRV group. (XLSX)
