# Supplementary figures and images for: Distinct transcriptional modules in the peripheral blood mononuclear cells response to human respiratory syncytial virus or to human rhinovirus in hospitalized infants with bronchiolitis
Source: PLoS One. 2019 Mar 7;14(3):e0213501. doi: 10.1371/journal.pone.0213501 (PMC6405118; doi:10.1371/journal.pone.0213501)

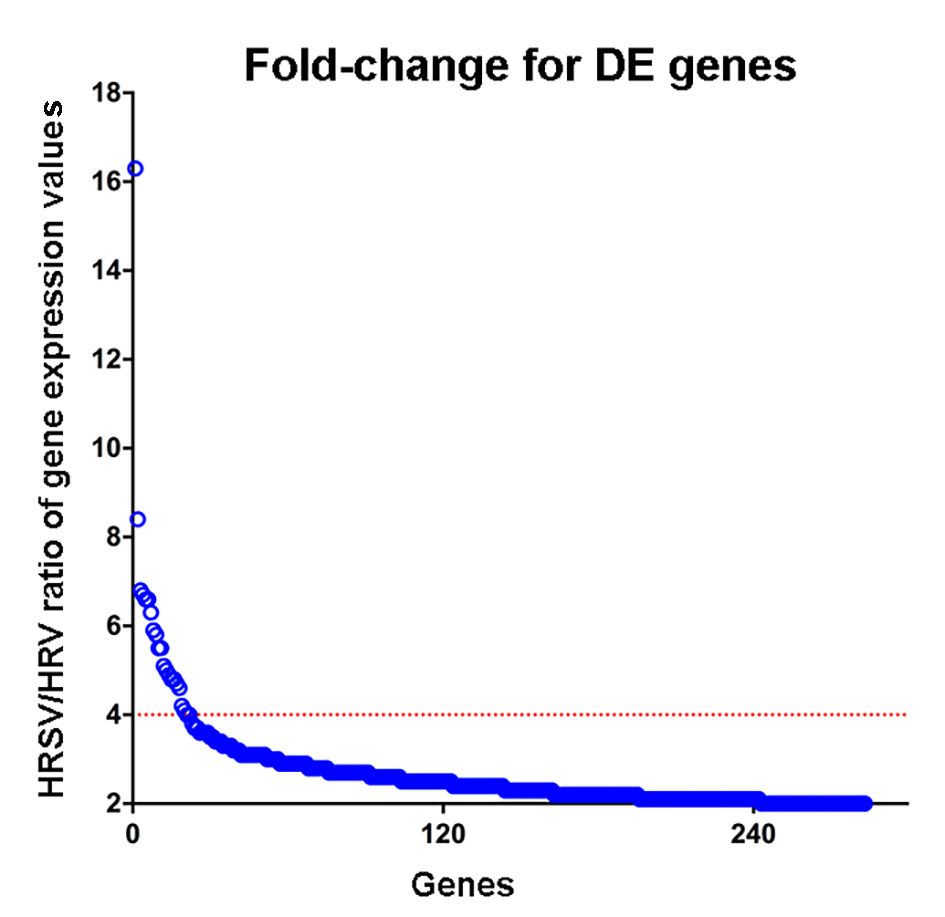

Supplement: S1 Fig — Twenty-two genes were identified as candidate gene markers. The red dot line indicates the cut off (fold-change 4.0) adopted here to consider highly differentially expressed genes. (TIF) [file pone.0213501.s001.tif]

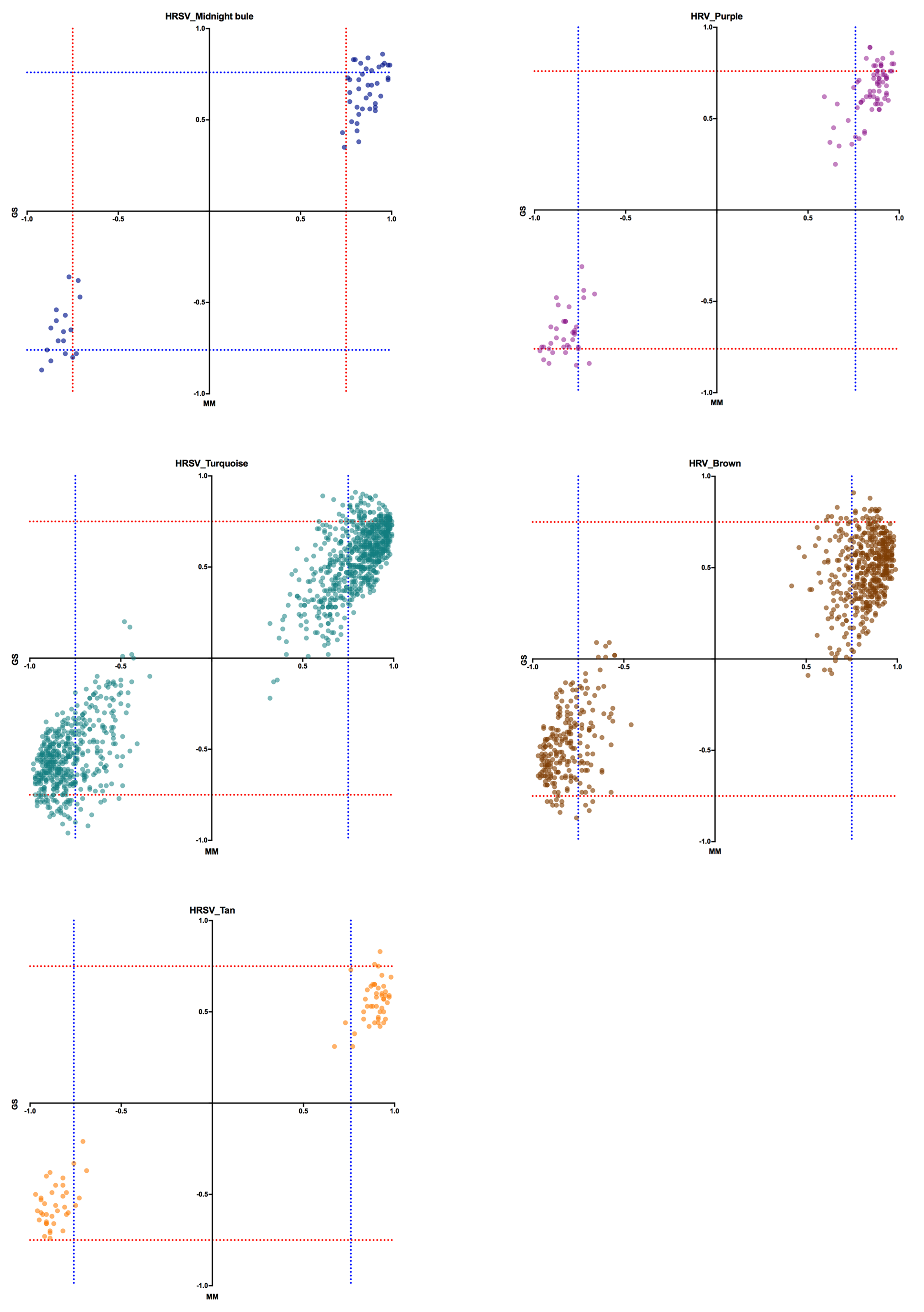

Supplement: S2 Fig — Scatterplots between MM (x-axis) and GS (y-axis) of the genes in the i) midnight blue, turquoise, and tan modules of the HRSV group; ii) brown and purple modules of the HRV group. The red or blue dot lines indicate, respectively, the cut off of GS or MM values significantly for HRSV or HRV groups (p < 0.01). (TIF) [file pone.0213501.s002.tif]
